# Interaural correlation of acoustic hearing preservation following sequential cochlear implantation

**DOI:** 10.3389/fneur.2025.1629333

**Published:** 2026-01-12

**Authors:** Armine Kocharyan, Carolina Chu, Rustin Kashani, Rachel A. Scheperle, Douglas M. Bennion, Sarah Coleman, Eva Rasche, Jacob J. Oleson, Bruce J. Gantz, Camille C. Dunn, Marlan R. Hansen

**Affiliations:** 1 House Ear Clinic, Los Angeles, CA, United States; 2 Department of Otolaryngology-Head and Neck Surgery, University of Iowa Hospitals and Clinics, Iowa City, IA, United States; 3 Department of Otolaryngology, Emory University Hospital, Atlanta, GA, United States; 4 Department of Biostatistics, Carver College of Medicine, University of Iowa, Iowa City, IA, United States

**Keywords:** hearing, cochlear implantation, sequential cochlear implantation, inflammation, immune, hearing preservation

## Abstract

**Introduction:**

Compared to unilateral cochlear implantation, bilateral cochlear implantation demonstrates multiple advantages in speech understanding in noise and sound localization, especially when acoustic hearing is preserved in both ears. However, outcomes and factors influencing acoustic hearing preservation following cochlear implant (CI) remains poorly understood. Patients receiving sequential cochlear implantation are a valuable population to investigate as studying bilateral ears allows for a high degree of control of individual factors that may be affecting trends in hearing.

**Methods:**

This is a retrospective analysis of prospectively collected data. Twenty-three subjects met criteria for CI with residual hearing, defined as low frequency (LF) pure tone average (PTA) of 65 dB or less at 125, 250, and 500 Hz, and received sequential cochlear implantation were included in the study. Audiometric data was collected on all subjects pre/post-operatively. Speech perception was assessed in everyday listening conditions pre/post-implantation using CNC and AzBio materials.

**Results:**

Bilateral hearing preservation was achieved in 56.5% (13/23) of patients. Of the 16 patients with hearing preservation following the first implant, 81% (13/16) of them achieved bilateral hearing preservation following the second implant in the contralateral ear. Speech understanding scores improved after sequential cochlear implantation (*p* = 0.019). There was no significant correlation between length of electrode array and LFPTA in either ear (*p* = 0.464 and *p* = 0.699). About half (49%) of subjects retained functional acoustic hearing in the initially implanted ear 11 years post-implantation. There was significantly decreased retention rate of hearing preservation in the second ear (*p =* 0.014). Age at implantation overall significantly associated with loss of hearing preservation (*p* = 0.040).

**Discussion:**

Hearing preservation following sequential cochlear implantation improved everyday listening abilities despite greater loss of acoustic hearing in the sequentially implanted ear. Age at implantation was the only factor observed to correlate with hearing preservation over time. Given known histopathological evidence of inflammation following cochlear implantation, it is likely that an immune mediated process may be responsible for delayed and greater loss of hearing in the sequentially implanted ear. Therefore, individuals receiving sequential cochlear implantation are an excellent population in future studies investigating the inflammatory and immunomodulatory mechanisms that may be responsible for delayed hearing loss following cochlear implantation.

## Introduction

Over the last decade, a growing trend has emerged in the adoption of Electric Acoustic Stimulation (EAS), also known as hybrid cochlear implantation, among individuals with profound high-frequency hearing loss and relatively intact low-frequency hearing (1). This trend reflects the superior performance of EAS compared to conventional, electric only stimulation (2, 3). Compared to EAS, the diminished performance of a conventional cochlear implant (CI) primarily stems from decreased frequency resolution capabilities (4–8) along with loss of temporal fine structure encoding (9). Exclusive reliance on electrical stimulation presents challenges in effectively discerning acoustic signals that are essential for speech-in-noise segregation, music melody and timbre recognition, pitch perception, and emotional discernment of speech through detection of speech prosody (10–12). Given the significant benefit that preservation of acoustic hearing provides, various approaches have emerged to mitigate against hearing loss including soft surgical techniques (13, 14), design of electrode arrays to be shorter and thinner (12, 15–19), drug elution (20, 21), intraoperative acoustic monitoring (e.g., electrocochleography) (22–25), and even the use of robotics-assisted insertion (26, 27).

Considerable variability exists in the extent of hearing preservation, and the temporal dynamics of low frequency hearing loss after cochlear implantation differ. Though some individuals initially retain acoustic hearing immediately after cochlear implantation, a multitude of individuals experience varying degrees of delayed onset/long-term hearing loss, beyond 3 months post-implantation (1). Immediate hearing loss can be predominantly attributed to cochlear trauma induced by electrode array insertion and related acute inflammatory response (1). Factors contributing to delayed onset acoustic hearing loss have remained largely elusive (28, 29), but demographic variables such as the age at implantation (30–33) and chronic inflammation and fibrosis of the cochlea (34, 35), perhaps reflected by increased electrode impedances (36, 37), may play a role. In conjunction with these observations, it is thought that an immune-mediated and/or inflammatory mechanism may underlie the trends in hearing outcomes observed in patients who receive cochlear implantation especially given the variability in outcomes among individuals over time. Therefore, further investigation of factors such as gender, age, and electrode array length and their interplay with inflammatory factors may aid understanding of the functional effects of cochlear implantation on acoustic hearing in the larger population over time.

Sequential, bilateral cochlear implantation, defined as initial cochlear implantation followed by cochlear implantation in the contra-lateral ear after a given time period, has demonstrated notable advantages when compared to traditional unilateral CI including improved speech understanding in noise and enhanced sound localization (38, 39). Likewise, bimodal hearing, defined as unilateral CI with hearing aid (HA) in the non-implanted ear, also provides significant benefit compared to unilateral CI (39). Importantly, there are limited reported outcomes regarding acoustic hearing preservation following sequential cochlear implantation and the impact of sequential cochlear implantation on hearing preservation patterns across both ears remains unknown. Furthermore, the factors influencing hearing preservation after sequential cochlear implantation remain poorly understood. In this report, we present the results of an ongoing prospective study aimed at shedding light on outcomes and factors associated with hearing preservation following sequential cochlear implantation. Furthermore, we also demonstrate the great potential that studying this unique population has in further investigating immune mediated and inflammatory mechanisms that may impact hearing overall following cochlear implantation.

## Materials and methods

### Study design and subject selection

This is a retrospective analysis of prospectively collected data. All research procedures adhered to the ethical guidelines of the protection of human subjects and received approval from the University of Iowa Institutional Review Board (IRB #201110703). All patients consented to participate in this study and to second cochlear implantation.

A total of 23 individuals participated in the study, comprising of 9 males and 14 females. Inclusion criteria were post-lingually identified adults who met the Food and Drug Administration (FDA) criteria for cochlear implantation and exhibited residual low frequency hearing, which was defined as a low frequency pure-tone average (LFPTA) of less than 65 dB at 125, 250, and 500 Hz. The data presented represent the “most recent” documented data collection. The mean “most recent” postoperative test timepoint documenting amount of cochlear implantation experience for each ear was 97 ± 11.02 months for the first ear implanted and 23 ± 3.76 months for the second implanted ear.

### Device selection and surgical approach

The first ears of all subjects were implanted within the date range of 11/2004 and 03/2022. Sequential implants were performed within the date range of 02/2017 and 08/2022. CI systems were selected at time of evaluation for individual patients through shared decision making and influenced by device options that were available at any given time point. Devices represented in this sample include Cochlear (L24, S8, 422/522/624), Med-EL Flex (20, 24, 28), and Advanced Bionics Ultra Slim J. All devices were implanted by one of two surgeons (MRH and BJG) employing soft surgical techniques as described previously (40) with slow insertion and administration of 10 mg of intra-operative dexamethasone. A portion of patients who received their surgeries between 2009 and 2019 also received oral prednisone for 1 week post-operatively and then again for 1 week starting the day prior to CI activation. All but two patients received full insertions. One patient had 1 electrode left outside of the cochlea in both the first and second cochlear implant. The second patient had 2 electrodes left outside of the cochlea in the second cochlear implant.

### Audiometric and speech recognition data collection

Audiometric pure-tone air conduction thresholds at 125, 250, and 500 Hz were included in this analysis. Functional postoperative hearing preservation was defined as air-conduction thresholds <85 dB HL for each individual frequency at 125, 250, and 500 Hz. Audiometric testing was completed prior to implantation, at initial activation, and at every follow-up appointment, which was typically scheduled at 1-month, 3-, 6-, and 12-months, and annually over time.

Speech perception scores using the Consonant-Nucleus-Consonant (CNC) Word Test and AzBio Sentences were presented at 60 dB A at 0-degrees azimuth. CNC words were presented in quiet, and AzBio Sentences were presented in a + 5 dB signal-to-noise ratio with the noise emanating from the front. Subjects were tested preoperatively and postoperatively at 3-, 6-, and 12-months postoperatively and longitudinally thereafter at various times. Subjects were tested in their everyday listening condition, which is defined as the hearing condition that they used on a daily basis (41, 42).

Of note, some subjects were unable to attend all postoperatively scheduled appointments due to scheduling conflicts, sickness, or travel distance associated with multiple appointments. Additionally, due to time constraints during some attended participant postoperative appointments, not all data was able to be collected. Data presented in this manuscript are retrospective in nature and represents data collected from associated appointments.

### Statistical analysis

#### Individual hearing preservation outcomes

Hearing preservation outcomes were assessed by comparing the change in low-frequency pure-tone average (LFPTA) at 125, 250, and 500 Hz preoperatively to postoperatively in the first implanted ear versus the second implanted ear. A paired *t*-test was utilized to analyze for any significant difference in pre-operative hearing between both ears. We first computed the change in LFPTA for the second implanted ear from baseline preoperative measurement to most recent measurement and then the change in LFPTA for the first implanted ear was computed over the same time interval. For each ear, the change from pre-operative values was evaluated using a paired *t*-test. To determine whether the amount of change in LFPTA was greater for Ear #2 versus Ear #1, another paired *t*-test was used.

We included an analysis to determine if the length of electrode array is related to hearing preservation outcomes. Using a paired *t*-test, we examined LFPTA separately for those sequential users having electrode arrays that were the same length, and then sequential users that had longer electrode arrays in Ear #2. The null hypothesis for this test was that there was no difference in hearing loss in Ear #1 and Ear #2. A Wilcoxon signed rank test with continuity correction achieved the same results and thus findings of the paired *t*-test are presented.

#### Longitudinal hearing outcomes across both ears

Speech perception using the subjects’ every day listening configuration was first evaluated and assessed via a paired *t*-test comparing pre-sequential implantation listening scores to post- sequential implantation scores. Then speech perception in CI users and those with hearing preservation were compared within each time point using Welch’s two sample *t*-tests. Recipients prior to sequential cochlear implantation were grouped together by those who initially had unilateral hearing preservation and those that did not. Results after sequential cochlear implantation were grouped together by those who had bilateral hearing preservation and those that did not.

#### Long term outcomes of hearing preservation over time

First, a Kaplan Meier plot was made to compare hearing preservation over time between Ear #1 and Ear #2. A marginal cox proportional hazards model was then fit to the data to formally assess association between time-to-event in Ear #1 vs. Ear #2 with additional covariates for gender and implant age included to adjust for possible confounders. A clustering term was included to adjust for the dataset having two survival outcomes per recipient.

## Results

### Patient demographics

Patient demographics including ages of implantation, dates of implantation, etiology of hearing loss, medical comorbidities, and types of CI models utilized are depicted in Table 1. Distribution of CI models for the first CI among subjects were: L24 (9 patients), S8 (4 patients), 422/522/624 (4 patients), Flex (4 patients), and SLIMJ (2 patients). Distribution of CI models for the sequential or second CI were: L24 (6 patients), 422/522/624 (11 patients), Flex (4 patients), and SLIMJ (2 patients) (Table 1). Electric hearing is denoted as CI, acoustic hearing is denoted as HA, and hearing preservation is denoted as CI+HA. After the first CI procedure, seven patients had electric hearing in one ear and acoustic hearing in the other, denoted as CI/HA, and 16 patients had unilateral hearing preservation in the implanted ear and acoustic hearing in the contralateral ear, denoted as CI+HA/HA. The mean age at the time of first implantation was 48.5 ± 17.0 years and the mean age at the time of second implantation was 54.6 ± 18.5 years, with an average duration of 6.2 ± 3.5 years between the 2 CI surgeries. Two subjects did not have enough postoperative LFPTA data collected for analysis at our time intervals of observation and were excluded from the portions of the study looking at LFPTA changes over time but did have enough speech recognition data to be included in analysis regarding CNC and AzBio scores.

**Table 1:** Patient demographics including etiology of hearing loss and medical comorbidities along with overview of the cochlear implant device type and relative length of sequential implant compared to initial implant in participants of the study.

Patient ID	Sex	Ear implanted first	Age (yr) and date of first cochlear implantation	CI type	Ear implanted second	Age and date of second CI implantation	CI type	Length difference of sequential vs initial CI	Etiology	Comorbidities
1	M	R	66.9, 02/2015	Nucleus CI422	L	73.2, 05/2021	Nucleus 624	Shorter	Chemotoxicity	Lung cancer, cardiovascular disease
2	M	R	47.3, 10/2014	Nucleus CI422	L	54, 06/2021	Nucleus 624	Shorter	Unknown	None
3	F	R	34.2, 12/2016	Nucleus CI522, straight electrode array	L	38.2, 12/2020	Nucleus 624	Shorter	PTEN hamartoma syndrome associated HL	None
4	F	L	33.4, 11/2014	Nucleus Hybrid L24	R	38.1, 07/2019	Nucleus Hybrid L24	Same	Autoimmune	None
5	M	L	18, 08/2014	Nucleus Hybrid L24	R	22.4, 01/2019	Nucleus Hybrid L24	Same	Congenital	None
6	F	R	53.4, 08/2014	Nucleus Hybrid L24	L	57.5, 09/2018	Nucleus Hybrid L24	Same	Unknown	None
7	M	R	19.1, 03/2022	Clarion HiRes Ultra Slim J	L	19.2, 05/2022	Clarion HiRes Ultra Slim J	Same	GJB2-related HL	None
8	F	R	44.6, 09/2018	Clarion HiRes Ultra Slim J	L	48.2, 04/2022	Clarion HiRes Ultra Slim J	Same	Unknown	None
9	F	R	31.6, 10/2012	Med-El Synchrony Flex 24	L	37.8, 01/2019	Med-El Synchrony Flex 24	Same	Congenital	None
10	M	L	82.8, 01/2018	Med-El Synchrony Flex 24	R	86.2, 06/07/2021	Med-El Synchrony Flex 24	Same	Unknown	CAD, HLD
11	M	R	19.4, 12/2020	Nucleus 624	L	20.4, 12/20/2021	Nucleus 624	Same	TMPRSS3 (DFNB8/12) associated SNHL	None
12	F	R	54.4, 05/2010	Nucleus Hybrid L24	L	65, 12/10/2020	Nucleus 624	Longer	Unknown	T2DM, HLD
13	F	R	48.9, 07/2014	Nucleus Hybrid L24	L	53.9, 07/22/2019	Nucleus CI522, straight electrode array	Longer	Unknown	SSNHL of L ear
14	M	L	57.7, 01/2014	Nucleus Hybrid L24	R	65.4, 10/2021	Nucleus 624	Longer	Unknown	None
15	F	R	50.8, 03/2015	Nucleus Hybrid L24	L	57.2, 08/2021	Nucleus 624	Longer	Familial	Cardiovascular disease
16	F	L	53.5, 12/2006	S8	R	63.9, 05/2017	Nucleus Hybrid L24	Longer	Unknown	None
17	F	R	55.7, 05/2006	S8	L	70, 09/2020	Nucleus 624	Longer	Unknown	HTN
18	F	R	50.1, 09/2005	S8	L	61.8, 05/2017	Nucleus Hybrid L24	Longer	Autoimmune	HLD
19	M	R	51.5, 11/2004	S8	L	63.8, 02/2017	Nucleus Hybrid L24	Longer	Unknown	OSA
20	M	R	67.6, 10/2017	Med-El Synchrony Flex 20	L	72.4, 08/2022	Med-El Synchrony Flex 24	Longer	Unknown	HLD, OSA
21	F	R	75.2, 08/2016	Nucleus Hybrid L24	L	80.2, 08/2021	Nucleus 624	Longer	Meniere’s disease	None
22	F	L	58.1, 09/2015	Nucleus Hybrid L24	R	62.8, 06/2020	Nucleus 624	Longer	Noise associated	HLD
23	F	R	40.8, 03/2017	Med-El Synchrony Flex 24	L	45.3, 09/2021	Med-El Synchrony Flex 28	Longer	Unknown	Mitral valve prolapse

### Hearing preservation outcomes

**Figure 1:**
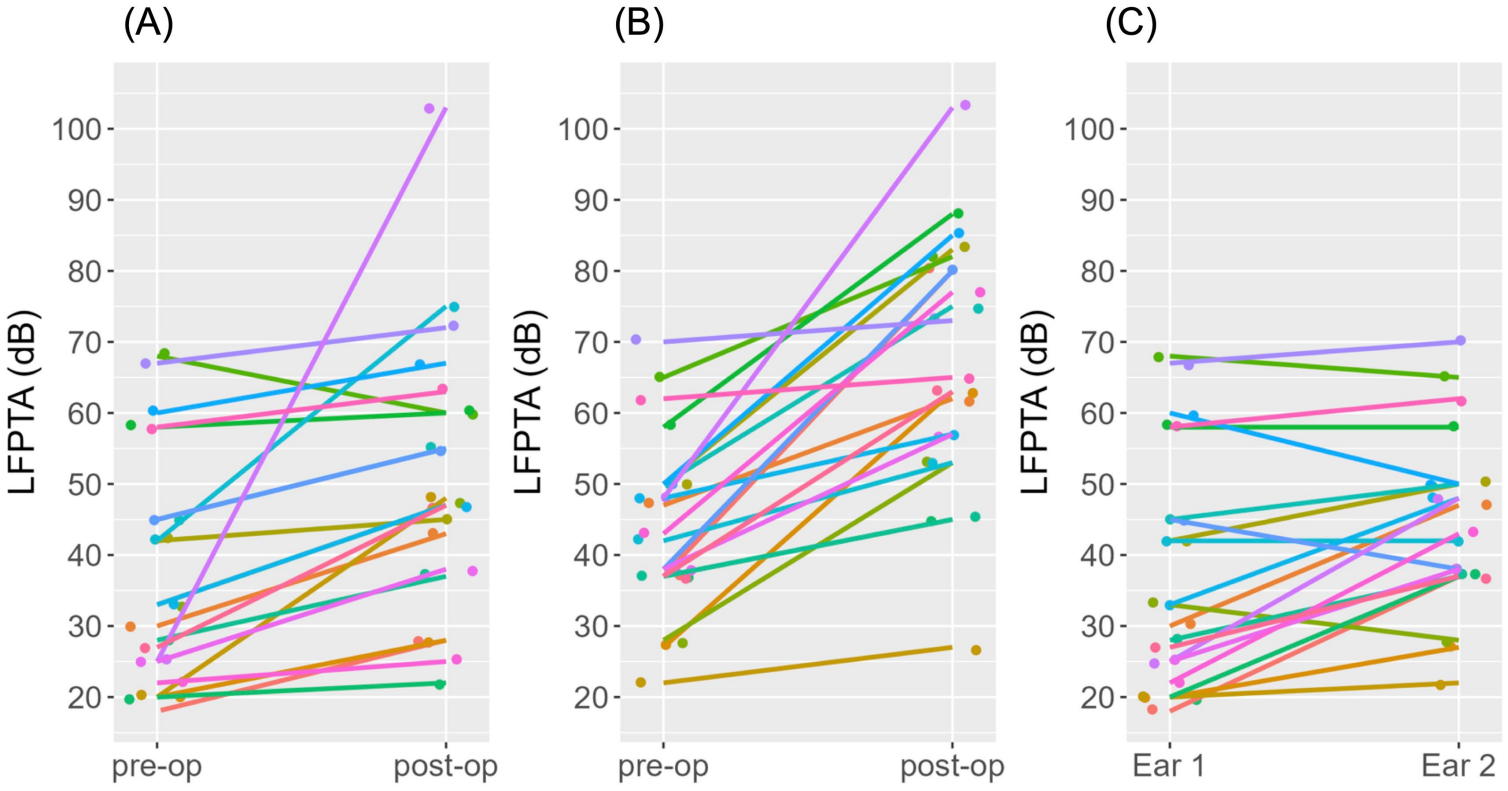
Line graphs displaying LFPTA measurements for each individuals’ ears prior to first and second cochlear implantation. **(A)** Depicted here are LFPTA measurements for each individual’s Ear #1 before and after first cochlear implantation. **(B)** Depicted here are LFPTA measurements for each individual’s Ear #2 before and after sequential cochlear implantation. **(C)** Depicted here is a comparison of LFPTA measurements of each individual’s Ear #1 prior to first cochlear implantation and Ear #2 prior to second cochlear implantation.

**Figure 2:**
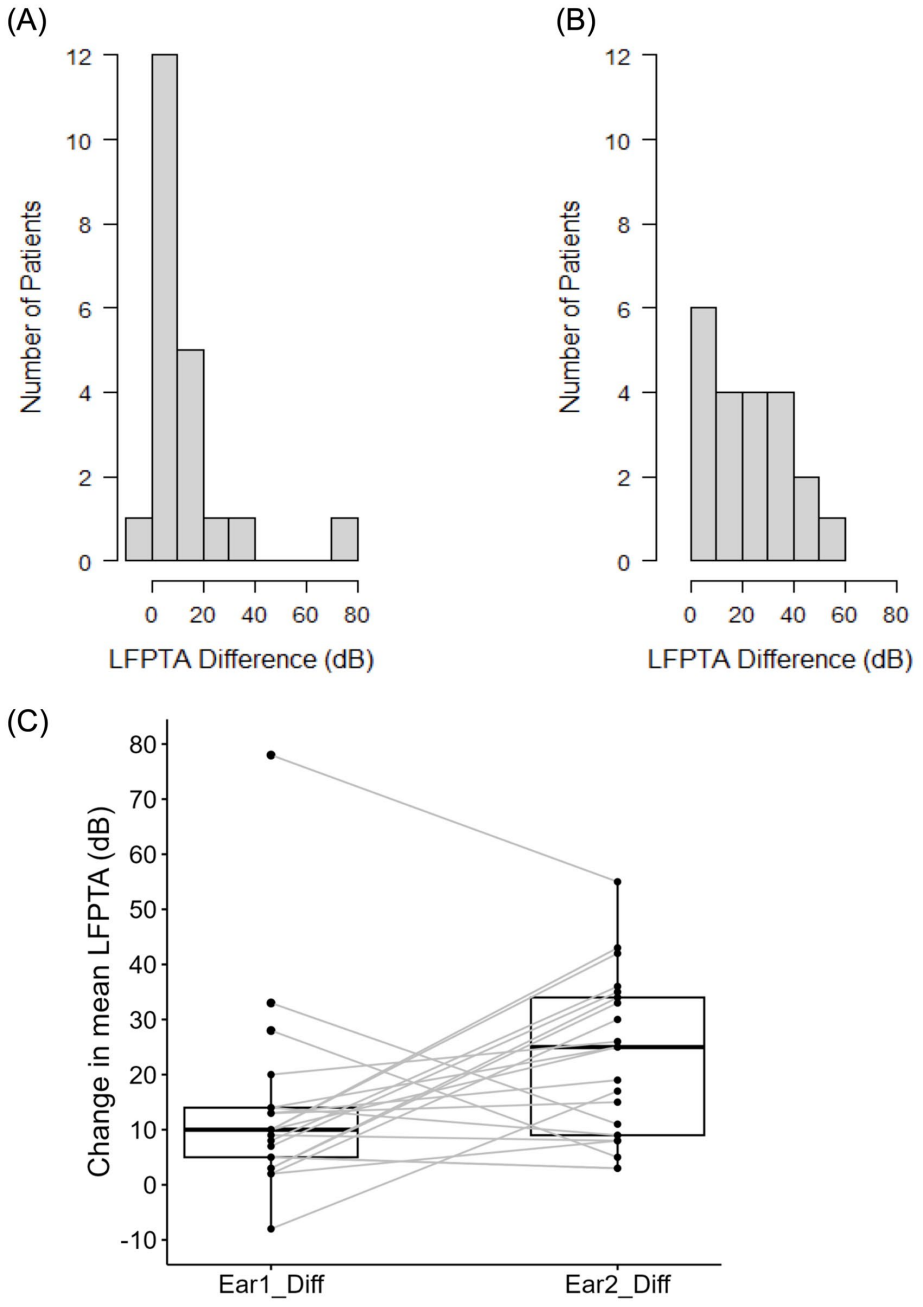
Bar graphs displaying LFPTA differences in Ear #1 and Ear #2 and box and whisper plot comparing the LFPTA change between Ear #1 and Ear #2. **(A)** The mean LFPTA change in Ear #1 was 13.29 dB. **(B)** The mean LFPTA change in Ear #2 was 22.95 dB. **(C)** There is a significantly larger change in mean LFPTA of Ear #2 compared to Ear #1 (*t* = −2.36, df = 20, *p* = 0.028).

Table 1 shows the device type used for all 23 participants in the study categorized by length of electrode array relative to original CI. Pre- and post-operative audiograms for both ears are depicted in Figures 1A,B. Mean pre-operative LFPTA for Ear #1 was 37.43 ± 16.51 dB and for Ear #2 was 44.48 ± 12.41 dB. Mean post-operative LFPTA for Ear #1 was 50.71 ± 19.25 dB and for Ear #2 was 67.43 ± 17.74 dB. There was significant hearing loss in both Ear #1 (*t* = 3.51, df = 20, *p* = 0.002) and Ear #2 (*t* = 7.08, df = 20, *p* < 0.001). There was a significant difference in pre-operative LFPTA between Ear #1 and Ear #2 (*t* = −3.41, df = 20, *p* = 0.003) with average pre-operative hearing in Ear #2 7.05 dB higher than the average pre-operative hearing in Ear #1 (Figure 1C). When comparing the change in Ear #1 versus Ear #2, the degree of low frequency hearing loss was significantly higher (*t* = −2.36, df = 20, *p* = 0.028) in the ear that was implanted second (22.95 ± 14.86 dB), compared to the ear that was implanted first (13.29 ± 17.34 dB) (Figure 2) as depicted by the larger LFPTA changes in the ear implanted second.

**Figure 3:**
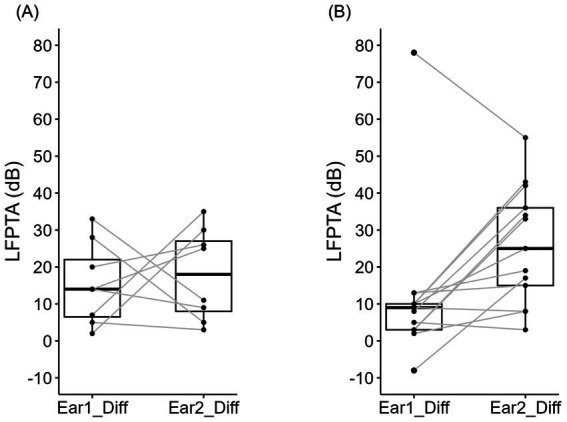
Box and whisker plots comparing hearing loss following first and second cochlear implantation, separated by subjects who received the same electrode array **(A)** or a longer electrode array **(B)** in the second ear. **(A)** There is no significant difference in LFPTA between Ear #1 and Ear #2 in those who received sequential cochlear implantation with the same electrode array length (*p* = 0.717). **(B)** There is a significantly greater LFPTA change in Ear #2 when compared to Ear #1 (*p* = 0.014) in those who received a longer electrode array length in sequential CI.

Because the risk of intracochlear structural damage increases with insertion depth, and because a higher proportion of longer electrode arrays were used for sequential implants compared to initial implants, the potential influence of electrode array length was evaluated. Twelve of 23 subjects received a longer electrode array in the 2nd ear to be implanted compared to the first. At the group level, there was no significant difference in average LFPTA change between Ear #1 (15.38 ± 11.03) and Ear #2 (18.00 ± 12.36) in those who received sequential cochlear implantation with the same electrode array length (*t* = −0.38, df = 7, *p* = 0.717) (Figure 3A). However, in patients who received a longer electrode array during sequential cochlear implantation, Ear #2 (26.00 ± 15.86) did show a significantly greater LFPTA change on average when compared to Ear #1 (12.00 ± 20.63) (*t* = −2.89, df = 12, *p =* 0.014) (Figure 3B). There was no statistically significant difference in pre-operative hearing in either Ear #1 (*t* = 0.88, df = 16, *p* = 0.395) or in Ear #2 (*t* = −0.30, df = 13, *p* = 0.769) of individuals who received the same or different array length for both ears.

### Hearing outcomes across both ears

**Figure 4:**
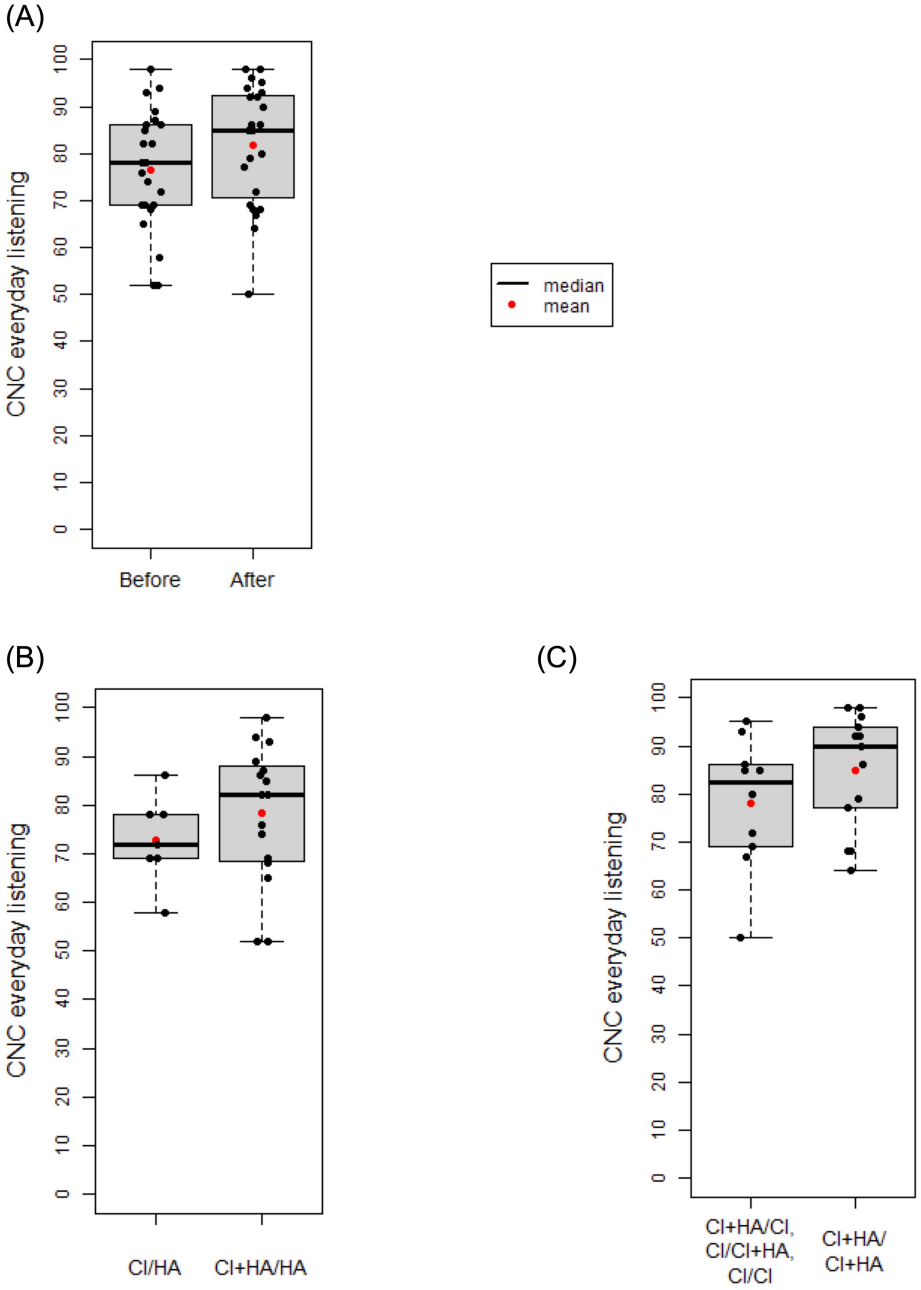
Box and whisker plots comparing mean CNC scores in those with (CI+HA) and without (CI) hearing preservation before and after sequential cochlear implantation. **(A)** There was a significant improvement in CNC with a mean change of 5.3 after sequential cochlear implantation (*t* = −2.54, df = 22, *p* = 0.019). **(B)** Prior to sequential cochlear implantation, the mean CNC score for those without hearing preservation (CI/HA) was 72.86. The mean CNC score for those with unilateral hearing preservation (CI+HA/HA) was 78.25. There was no significant difference observed between these two groups (*p* = 0.282). **(C)** After sequential CI, the mean CNC score for those with bilateral hearing preservation (CI+HA/CI+HA) was 84.7. The mean CNC score for those without bilateral hearing preservation was 78.2. There was no significant difference between these two groups (*p* = 0.248).

**Figure 5:**
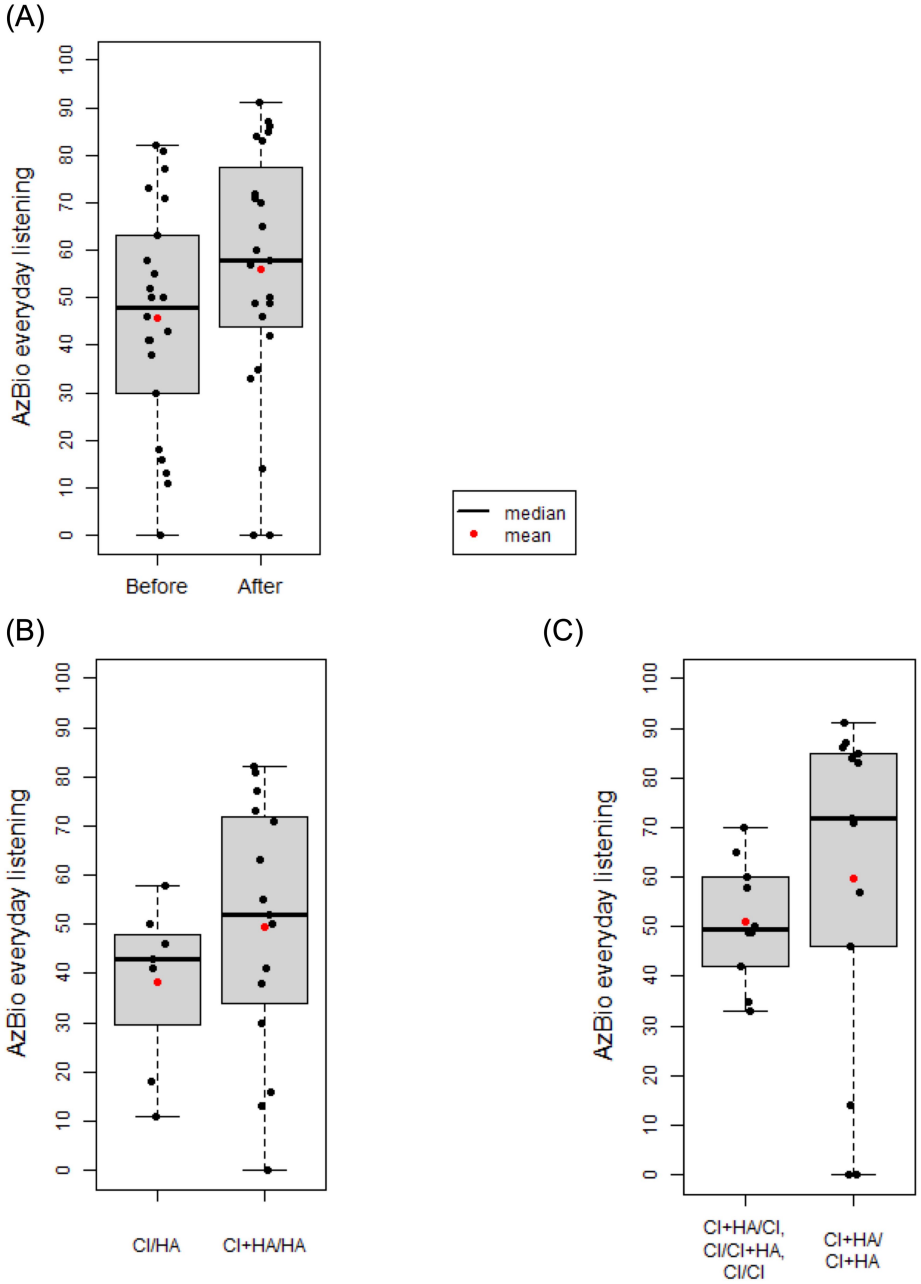
Box and whisker plots comparing mean AzBio scores in those with (CI+HA) and without (CI) hearing preservation before and after sequential cochlear implantation. **(A)** There was no significant different in AzBio scores after sequential cochlear implantation with a mean change of 8.7 (*t* = −1.93, df = 21, *p* = 0.067). **(B)** Prior to sequential cochlear implantation, the mean AzBio score for those without hearing preservation (CI/HA) was 38.14. The mean AzBio score for those with unilateral hearing preservation (CI+HA/HA) was 49.47. There was no significant difference observed between these two groups (*p* = 0.242). **(C)** After sequential CI, the mean AzBio score for those with bilateral hearing preservation (CI+HA/CI+HA) was 51.10. The mean AzBio score for those without bilateral hearing preservation was 59.69. There was no significant difference between these two groups (*p* = 0.412).

Table 2 depicts overall hearing preservation outcomes of all participants. Of the 7 patients with initial CI/HA, 4/7 (57%) patients were able to achieve unilateral hearing preservation CI (CI/CI+HA) and the remaining 3/7 (43%) patients had bilateral electric hearing only (CI/CI). Of the 16 patients with initial CI+HA/HA, 13/16 (81%) patients were able to achieve bilateral hearing preservation (CI+HA/CI+HA) and the remaining 3/16 (19%) patients continued to have only unilateral hearing preservation (CI+HA/CI) (Table 2). Overall, bilateral hearing preservation was achieved in a total of 13/23 (56.5%) of the patients. The paired *t*-test comparing pre-sequential implantation listening scores to post-sequential implantation scores determined that CNC significantly improved on average after sequential CI (*t* = −2.54, df = 22, *p* = 0.019) with a mean change of 5.3 (Figure 4A). When looking at each time point individually, prior to sequential CI, the mean CNC score for those without hearing preservation (CI/HA) (72.86 ± 8.91) was lower than the mean CNC score for those with unilateral hearing preservation (CI+HA/HA) (78.25 ± 14.02) though not at a statistically significant level (*p* = 0.282) (Table 2; Figure 4B). After sequential CI, the mean CNC score for those with bilateral hearing preservation CI (CI+HA/CI+HA) was 84.77 ± 12.19 while the mean CNC score for those with only unilateral (CI+HA/CI, CI/CI+HA) or no hearing preservation (CI/CI) was 78.20 ± 13.75. There was no significant difference between these two groups (*p* = 0.248) (Table 2; Figure 4C). Similarly, prior to sequential CI, the mean AzBio score was lower (38.14 ± 17.18) in those without hearing preservation (CI/HA) compared to those with unilateral hearing preservation (CI+HA/HA) (49.47 ± 26.09). There was no statistical difference between the two (*p* = 0.242) (Table 2; Figure 5B). After sequential CI, the mean AzBio score for those with bilateral hearing preservation (CI+HA/CI+HA) was 51.10 ± 12.28 compared to 59.69 ± 34.01 for those without bilateral hearing preservation. There was no significant difference between these two groups (*p* = 0.412) (Table 2; Figure 5C). Paired *t*-test comparing pre- and post-sequential implantation AzBio scores also showed no significant difference after sequential CI (*t* = −1.93, df = 21, *p* = 0.067) with a mean change of 8.7 (Figure 5A).

**Table 2:** Overall outcomes of hearing preservation prior to and after sequential cochlear implantation.

Initial CI hearing preservation features (Ear#1/Ear#2)	Mean CNC	Significance	Mean AzBio	Significance	Sequential CI hearing preservation features (Ear#1/Ear#2)	Mean CNC	Significance	Mean AzBio	Significance
7 participants with CI/HA	72.86	*p* = 0.282	38.14	*p* = 0.242	4 participants with CI/CI+HA	78.2	*p* = 0.248	51.10	*p* = 0.412
					3 participants with CI/CI				
16 participants with CI+HA/HA	78.25		49.47		3 participants with CI+HA/CI				
					13 participants with CI+HA/CI+HA	84.7		59.69	

^*^CI denotes electric hearing, HA denotes acoustic hearing, CI+HA denotes hearing preservation. Recipients were categorized as those who had unilateral hearing preservation initially (CI/HA) and those who had bilateral hearing preservation (CI+HA/CI+HA) after sequential cochlear implantation.

### Long term outcomes of hearing preservation over time

**Figure 6:**
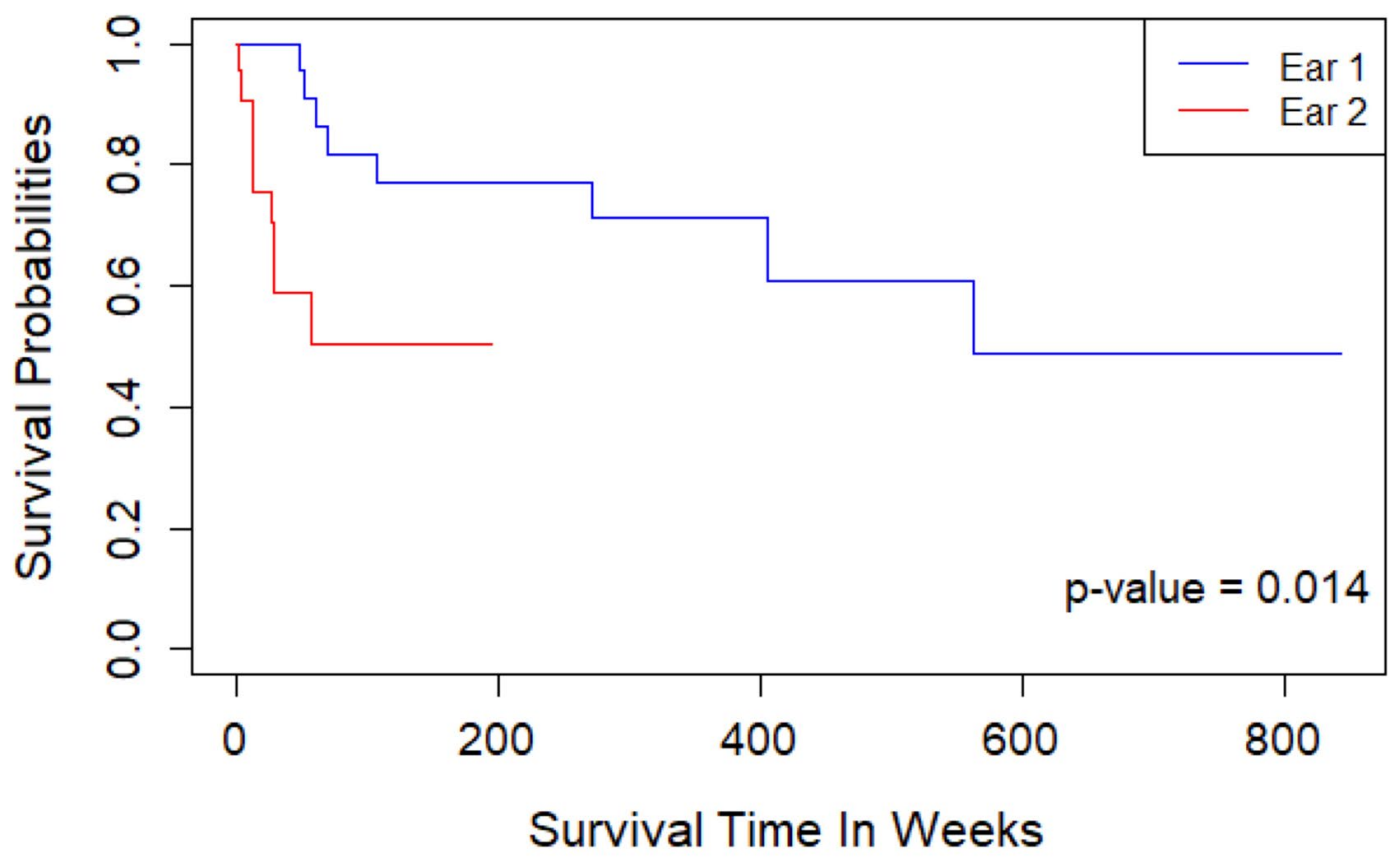
Kaplan Meier curve showing length of continued hearing preservation after first and second cochlear implantation. Ear #2 had a 2.79x higher hazard rate compared to Ear #1 (*p* = 0.014); a significantly decreased rate of retention of hearing preservation. Retention of hearing preservation for Ear #1 was 91% of patients 1 year after implantation, 82% after 2 years, 77% after 3 years, 71% after 6 years, 61% after 8 years, 61% after 10 years, and 49% of patient continuing to have acoustic hearing preservation 11 years after initial implantation. Retention of hearing preservation for Ear #2 was 76% at 6 months after surgery, 59% after 1 year, and 50% after 1.2 year.

Maintenance of hearing preservation/LF hearing (LFPTA<85 dB HL) over time is shown in Figure 6. After adjusting for gender and age at implantation, the second ear showed a 2.79x higher hazard rate compared to the first ear (*p* = 0.014), and a significantly decreased rate of retention of hearing preservation. Additionally, age at implantation was associated with increased hazard rate for hearing preservation CI with each yearly increase in age at implantation leading to a 1.038x greater hazard rate (*p* = 0.040). Moreover, the observed retention of hearing preservation for Ear #1 was robust with 91% of patients having acoustic hearing preservation 1 year after implantation, 82% after 2 years, 77% after 3 years, 71% after 6 years, 61% after 8 years, 61% after 10 years, and 49% of patient continuing to have acoustic hearing preservation 11 years after initial implantation. On the other hand, observed retention of hearing preservation for Ear #2 was less robust at 76% at 6 months after surgery, 59% after 1 year, and 50% after 1.2 year.

## Discussion

Herein, we present the outcomes of sequential cochlear implantation and report the rate and patterns of hearing preservation among participants over time. A major strength in our work was the study of bilateral ears. Participants thus served as a source of internal control, allowing for greater control over systemic factors that may influence differences in hearing between the two ears as the etiology for hearing loss is likely the same among bilateral ears. Moreover, variability among physiology of different individuals and comorbidities was also better controlled by comparing hearing preservation between bilateral ears of a single individual. Results showed that there was a greater amount of acoustic hearing loss after sequential cochlear implantation as evidenced by greater LFPTA change in Ear #2 and fewer individuals with functional residual acoustic hearing over time. In particular, there was a greater change in LFPTA of the sequentially implanted ear observed in those who received a longer electrode array during sequential cochlear implantation than initial cochlear implantation compared to those who received sequential cochlear implantation with electrode arrays of the same length. Initially, it was thought that a longer electrode array length could lead to more trauma during insertion, leading to a greater change in LFPTA in the second ear. Beyond greater risk of insertion trauma, a longer electrode array also introduces more foreign biomaterials into scala tympani and these materials coupled with the associated fibrous capsule displaces a greater volume of the perilymph compared to shorter electrode arrays. However, in this study, regression analysis of this observed association between longer array length and greater LFPTA change was found to be r = 0.0913, suggesting that electrode array length alone is not likely to fully account for the observed change in LFPTA. The unclear implication of this observed association is reflective of current studies demonstrating varying observations and opinions on hearing preservation over time in relation to electrode array length (43–45). Instead, other factors are suspected to play major roles in these data. For example, this study found a hazard rate for hearing loss after cochlear implantation that directly correlated with increased age at implantation. An analysis of a subset of the Hybrid S8 Food and Drug Administration trial likewise found increasing age as a risk factor for hearing loss after cochlear implantation (29). These observations are in line with other studies that showed superior hearing preservation in pediatric and adolescent patients compared to adult patients (30–33, 46–49), though, in these studies, there was variability among etiologies of hearing loss along with other factors that may have confounded those results.

Another consideration that could contribute to the observed greater LFPTA change in the sequentially implanted ear could be the immunogenicity of the cochlear implant biomaterials. Though the manufacturer for several of the CIs was the same, the reaction of each individual to the biomaterials is highly variable and therefore a potential factor for further investigation. As with any other implantation of foreign biomaterials, the body will have an immune reaction, and a fibrotic capsule is universally seen in cochleae implanted with a CI (50, 51). In one study, histopathologic features of temporal bones in patients with CIs showed that 75% had evidence of a foreign body reaction with a delayed hypersensitivity reaction suggested as a possible contributor to delayed CI failure (34). In another study, histopathology showed a granulomatous reaction to the electrode array in 96.4% of the temporal bones along with eosinophilic infiltration in 25% of the temporal bones (52). Moreover, this inflammatory response was significantly greater at the basal turn of the cochlea near the site of the cochleostomy, as seen from the greater degree of fibrosis in this area (52) and postulated to cause a damping effect on passive properties of the basilar membrane, compromising basilar membrane velocity and function (53). Importantly, this observed fibrosis has been shown not only to be due to the physiologic response to a foreign object but also highly dependent on the surgical technique employed during cochlear implantation (54–56), thus emphasizing subjective factors impacting patterns and timing of hearing loss. Several studies have shown that, in addition to contributing to a pro-fibrotic environment, cytokines in the inflammatory response also increase permeability of the blood-labyrinthine barrier, thereby disrupting the homeostasis of the inner ear environment (57–59). In turn, this disruption can further aggravate the release of additional inflammatory cytokines, perpetuating damage (57). The effect of these cytokines has also been shown to directly affect the microvasculature of the cochlea leading to ischemic damage and subsequent loss of hearing via capillary vasoconstriction and decreased blood flow (60, 61). Other acoustic pathologies with an inflammatory component, such as Meniere disease, have also been shown to have an increase in blood-labyrinthine barrier permeability (62) secondary to inflammatory cytokines that are seen similarly elevated after a trauma like cochlear implantation (51, 63–68), highlighting the role that inflammation may have on the development of a pathologic inner ear environment and consequent acoustic dysfunction.

Though understudied regarding cochlear implantation at this time, the process of immune mediated acute, chronic, and second-set rejection has been extensively seen and demonstrated in allograft transplants (69) and may provide further explanation for greater LFPTA change in the second ear. It is possible that exposure to the initial CI leads to immune sensitization to the material (70, 71), thus predisposing to a more robust immune response in the setting of a second CI. This could perhaps lead to a more acute and severe immune reaction to explain the higher LFPTA change in the sequentially implanted ear or a longer chronic immune mediated inflammatory reaction to account for delayed hearing loss. In fact, it has been shown that in cochleae that received sequential cochlear implantations, there is a greater and more rapid increase in electrode impedances in the ear that was implanted second (72) which could correlate with a greater immune response in the second ear. Furthermore, delayed loss of acoustic hearing after cochlear implantation has been associated with increased electrode impedance, particularly with a rise in access resistance, which is felt to represent increased fibrotic tissue near the electrodes (36, 37).

Upregulation of inflammatory cytokines such as TNF alpha have been directly observed in an implanted ear secondary to both insertional trauma and physiologic response (63, 73) but it has not been as well documented whether this inflammatory response also affects the contralateral, non-implanted ear. However, there has been documentation regarding the bilateral increase in blood-labyrinthine barrier permeability in Meniere disease despite only unilateral symptoms (62), indicating that an inflammatory response may have systemic effects or at least impact bilateral ears. If the immunologic and microvascular sequelae due to the inflammatory response after initial cochlear implantation persists bilaterally, it is conceivable that the environment in which the sequential cochlear implantation takes place is one that would predispose to a more rapid or severe inflammatory reaction. However, no definitive conclusions can be drawn at this time. Though histopathologic studies have reliably demonstrated the presence of fibrosis around the electrode array, there have been several observations that show a lack of a significant relationship between amount of fibrosis in the implanted ear and duration of implantation (61, 74), implying that severity of inflammation and ensuing fibrosis may not be linearly correlated with time. Further, though molecular therapy targeting implicated cytokines has shown initial benefit in attenuating trauma associated hearing loss after cochlear implantation (65–67), long term effects have not yet been adequately studied and this potential therapy has not been applied in the case of bilateral implantation and loss of hearing over time. Additionally, several studies in humans have not found significant correlation between word recognition scores and volume of fibrosis (61, 74, 75), thus necessitating elucidation of the role and clinical impact of fibrosis versus upstream inflammatory pathways that led to fibrosis and calling into question whether traditional immunomodulatory treatments will provide the expected long-term benefits on hearing preservation over time.

An interesting trend noted in this study was that speech understanding scores improved after sequential cochlear implantation in all patients despite greater loss of hearing in the sequentially implanted ear. This speech understanding score was further increased in patients with unilateral hearing preservation cochlear implantation, with the greatest speech understanding scores observed in those with bilateral hearing preservation sequential cochlear implantation. A factor that may explain this trend may be sustainability of acoustic hearing preservation as depicted in the decades-long follow up for hearing preservation in the initially implanted ear. These observations further highlight the importance of making every effort to preserve residual cochlear function when placing an electrode array (76). With acoustic preservation remaining robust for half the population at the time of cochlear implantation procedure, it is possible that participants were able to continue using the acoustic hearing in Ear #1 and further supplement that with the sequentially implanted ear. At present, there has not been adequate time elapsed to observe trends of acoustic hearing preservation maintenance in the sequentially implanted ear to determine what effect hearing preservation cochlear implantation may have on the initially implanted ear and subsequently on the speech understanding scores. This highlights the necessity of continuing to follow with these patients to identify trends in acoustic hearing preservation after cochlear implantation over time.

Limitations of this study include inherent bias with device selection, the relatively small sample size at a single institution, and shorter-term follow-up after cochlear implantation. The variable time between initial and sequential cochlear implantations among participants may create a potential age bias that can lead to difficult direct comparisons regarding sequential cochlear implantation benefit. It is important to continue to follow these patients to further assess the longevity and impact of hearing preservation sequential cochlear implantation. Strengths of this study include analyzing effects of sequential cochlear implantation in bilateral ears of a single participant and then comparing these trends with other participants. This provides some control for potential confounding factors, such as etiology of hearing loss, and accounts for individual medical co-morbidities that may have impacted sequential cochlear implantation effects. Additionally, performance of CIs at a single institution provided a more consistent surgical technique and hearing preservation protocol, thereby minimizing variability and effect of associated perioperative factors on CI results.

The potential contribution of immunologic mechanisms to hearing preservation outcomes is supported by growing translational evidence, which provides a plausible biological basis for the trends observed in this study. While direct assessment of immune involvement was beyond the scope of this analysis, our findings are hypothesis-generating and warrant further investigation. Ongoing work at our center aims to expand upon these preliminary observations and explore the underlying immunologic processes in greater depth. In support of this framework, recent work from our group (72) examined electrode impedance dynamics in sequentially implanted patients, testing the hypothesis that the first implant may “prime” the contralateral ear to exhibit a more robust response to subsequent implantation. The study demonstrated earlier and greater increases in electrode impedance in the second implanted ear, findings consistent with a more pronounced immune response.

Historically, cochlear implant recipients were counseled to expect complete loss of residual acoustic hearing. However, the introduction of thin, flexible electrode arrays and “soft” surgical techniques has substantially reduced insertion trauma, allowing for preservation of residual hearing in many cases. In this cohort, all implantations were performed by two surgeons following a uniform hearing preservation protocol that remained unchanged throughout the study period. Although the absolute difference in hearing loss between the first and second implanted ears was small, it reached statistical significance. In most patients, the etiology of hearing loss was unknown. A key strength of this study is that each patient effectively served as their own control, minimizing intersubject variability. While some variables are inherently difficult to control, analysis of the contralateral ear allowed for reduction of potential confounding factors.

Our findings do not support the notion that short electrode arrays are inherently superior for hearing preservation; rather, electrode array length alone does not fully account for the observed outcomes. Although this work is not mechanistic or translational in nature, the immunologic hypothesis—supported by histological evidence from human cochlear studies—offers a biologically plausible framework for interpreting these findings.

While some of the specific electrode array models included in this study are no longer commercially available, the underlying principles remain clinically relevant. Contemporary arrays, such as those from MEDEL, now range from 20 mm to 34 mm, allowing for tailored insertion depths based on patient anatomy and surgical goals. Furthermore, our center and others continue to employ partial insertion techniques when hearing preservation is a primary objective.

## Conclusion

Hearing preservation following sequential cochlear implantation improved everyday listening abilities in CI recipients despite a greater loss of acoustic hearing in the ear implanted second. At this stage, we have not yet observed correlation of device type or electrode array length with hearing outcomes between the initially implanted and sequentially implanted ear. However, we have observed a direct correlation between age at implantation and success of hearing preservation cochlear implantation. The lack of clearly discernible factors impacting success of hearing preservation following sequential cochlear implantation may be in part attributed to the diverse variations and nuances present across individuals in terms of how each patient’s medical history, etiology of disease, and lifestyle factors may impact their physiologic response to implantation. Moreover, histopathologic data of temporal bones following implantation along with data in our study showing greater loss of acoustic hearing in the ear implanted second point toward an immune mediated process at play, a process that too has great variation among individuals. Continued follow-up with these participants is warranted and would provide a greater understanding on factors that may impact effects and durability of hearing preservation sequential cochlear implantation over time. Most importantly, individuals who receive sequential cochlear implantation comprise a unique population in which the individuals themselves serve as an internal control for variable factors that may mask underlying trends in hearing across the general population. Further study of this population has great potential in better understanding the immune mediated and inflammatory mechanisms that may impact hearing preservation and more broadly, hearing outcomes after cochlear implantation.

## Data Availability

The original contributions presented in the study are included in the article/supplementary material, further inquiries can be directed to the corresponding author.
